# Specimens of Artificial Teeth

**Published:** 1853-07

**Authors:** 


### SPECIMENS OF ARTIFCIAL TEETH.

We see in the last number of the Western Lancet, notice of a full set of teeth, made by Mr. J. Payne, of Gallopolis, Ohio, for exhibition at the World's Fair. This set is done up on platinum plate, and according to Dr. Allen's mode with continuous gum, and for neatness of execution, excel any thing of the kind we have ever seen. The upper as well as the lower set are rimmed inside and out, and arranged to give beauty and strength to the operation. The inner rim looks like a continuation of the plate, and keeps up the natural arch of the palate and receives the gum flown in on the palital face of the teeth, which looks like natures own "handy work." If the World's Fair shows prettier work, we should like to see it. Mr. Payne showed us also a second upper set done up in the same style. Mr. Payne was one of our students last session in the Ohio Dental College, and we noticed his remaining in the city sometime after the session closed. We can now explain the cause. We think his friends in Gallapolis will receive substantial service from his operations.

